# Artificial intelligence and machine learning for precision prevention of cognitive decline: integrating multimodal biomarkers, lifestyle interventions, and natural medicines from prediction to clinical practice

**DOI:** 10.3389/fnagi.2026.1924338

**Published:** 2026-08-05

**Authors:** Erika Smeriglio, Martina Imbesi, Jianbo Xiao, Antonella Smeriglio, Domenico Trombetta

**Affiliations:** 1 Department of Cognitive, Psychological and Pedagogical Sciences, and Cultural Studies (COSPECS), University of Messina, Messina, Italy; 2 Department of Chemical, Biological, Pharmaceutical and Environmental Sciences (CHIBIOFARAM), University of Messina, Messina, Italy; 3 Nutrition and Bromatology Group, Department of Analytical Chemistry and Food Science, Instituto de Agroecoloxía e Alimentación (IAA)-CITEXVI, Universidade de Vigo, Vigo, Spain; 4 Research Group on Food, Nutritional Biochemistry and Health, Universidad Europea del Atlántico, Santander, Spain

**Keywords:** artificial intelligence, clinical implementation, cognitive decline, machine learning, multimodal biomarkers, natural medicines, neurodegenerative diseases, precision prevention

## Abstract

Cognitive decline and neurodegenerative diseases are progressive, multifactorial conditions that may begin years before overt clinical diagnosis and reflect interactions among biological vulnerability, modifiable exposures, environmental determinants, and reduced brain resilience. This structured narrative review examines how artificial intelligence (AI) and machine learning (ML) can integrate clinical, biological, behavioral, and digital data to support earlier, personalized, and clinically actionable strategies for preserving cognitive health. Drawing on evidence from aging neuroscience, biomarker research, digital medicine, lifestyle prevention, natural-product pharmacology, and translational AI, we propose an AI-enabled framework for precision prevention and early management of cognitive decline. Within this framework, AI may support multimodal data integration, individualized risk prediction, digital phenotyping, biomarker-based stratification, intervention selection, natural-compound prioritization, and longitudinal monitoring. Lifestyle interventions and natural medicines are considered complementary components of personalized care whose value depends on biological plausibility, standardization, target engagement, and measurable cognitive or biomarker effects. However, translation from benchmark datasets to clinical practice remains limited by insufficient prospective and external validation, poor interpretability, dataset bias, limited generalizability, inadequate calibration and clinical-utility assessment, and incomplete integration into real-world workflows. Overall, AI may provide the integrative architecture needed to combine multimodal biomarkers, modifiable risk profiles, lifestyle interventions, and natural-product pharmacology within dynamic, person-centered precision-prevention pathways. Its clinical value will depend on transparent reporting, representative datasets, prospective evaluation, and demonstrable improvement in clinical decisions and patient outcomes.

## Graphical Abstract

**Figure:**
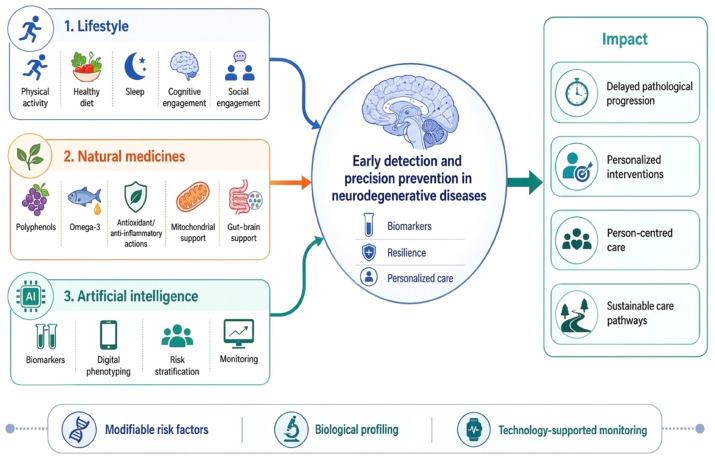
AI-enabled precision prevention of cognitive decline.

## 1 Introduction

Neurodegenerative diseases and dementia syndromes represent a major global public-health challenge. Dementia is characterized by progressive cognitive decline severe enough to compromise independence and may result from heterogeneous neurodegenerative and cerebrovascular conditions (American Psychiatric Association, 2022). Approximately 57 million people were living with dementia worldwide in 2021, with nearly 10 million new cases annually, and prevalence is projected to rise substantially by 2050 (World Health Organization, 2025). This growing burden, together with inequalities in access to diagnosis, specialist care, and long-term support, reinforces the need for earlier, scalable, and sustainable prevention-oriented strategies (World Health Organization, 2025; Alzheimer Europe, 2025a).

The National Institute on Aging–Alzheimer’s Association (NIA-AA) clinical criteria and research framework, including the amyloid, tau, and neurodegeneration [AT(N)] classification, marked a transition toward biologically informed models integrating clinical presentation with pathophysiological biomarkers (McKhann et al., 2011; Jack et al., 2018). This framework recognizes that neuropathological changes may develop years before overt symptoms, establishing a potential window for risk reduction, monitoring, and early intervention before extensive neuronal injury occurs. Subtle changes in cognition, affect, sleep, mobility, and daily functioning may also precede clinically evident decline, while minimally invasive blood-based biomarkers are increasing the feasibility of earlier and more scalable risk stratification (García-García et al., 2023; Teunissen et al., 2022).

In parallel, digital biomarkers derived from speech, gait, sleep, cognitive performance, smartphones, and wearable devices enable longitudinal assessment of behavioral and physiological changes in real-world settings (Kourtis et al., 2019; Lott et al., 2024). Together with biological, clinical, genetic, and lifestyle-related measures, these data create an increasingly multimodal assessment landscape. Artificial intelligence (AI) and machine learning (ML) offer computational approaches for integrating these heterogeneous sources, identifying latent patterns, and estimating individualized risks and disease trajectories that may not be captured by conventional single-marker analyses (Lee et al., 2024; Wang Y. et al., 2024; Cirincione et al., 2024). Their potential applications extend from diagnostic classification to progression prediction, patient stratification, longitudinal monitoring, and clinical decision support (Vaghari et al., 2025; Smeriglio, 2024a).

These developments support a transition from predominantly reactive, late-stage care toward earlier and more personalized strategies aimed at preserving brain health and strengthening resilience across the life course. Modifiable-risk reduction, biological profiling, lifestyle interventions, natural medicines, and technology-supported monitoring should therefore be considered complementary components of precision prevention rather than independent domains (Livingston et al., 2024; Lott et al., 2024). AI may provide the integrative layer through which these inputs are translated into individualized risk profiles, prioritized interventions, and adaptive monitoring pathways.

**FIGURE 1:**
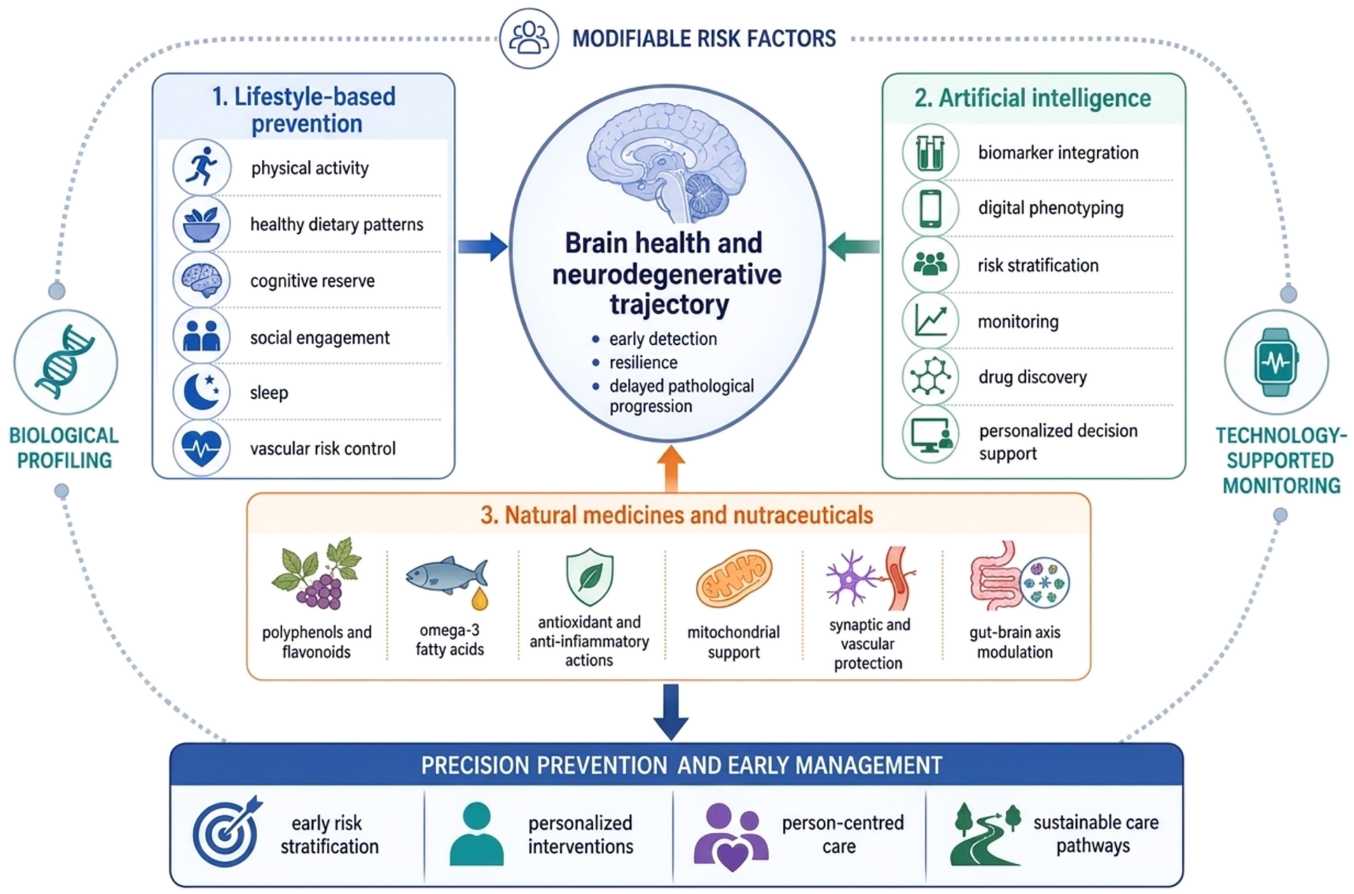
Artificial intelligence (AI)-enabled translational framework for precision prevention and early management of cognitive decline. Lifestyle-related risk factors, natural medicines and nutraceuticals, biological biomarkers, and digital monitoring are integrated through AI and machine-learning approaches to support early detection, individualized risk stratification, patient phenotyping, intervention selection, and longitudinal monitoring. The framework connects multimodal data integration with person-centered prevention and clinically actionable decision support. Created in BioRender.com.

Accordingly, this structured narrative review examines an AI-enabled translational framework for the precision prevention and early management of cognitive decline (Figure 1). Particular attention is given to multimodal risk prediction, biomarker-based and digital phenotyping, personalized lifestyle and multidomain interventions, AI-assisted natural-compound prioritization, and longitudinal evaluation of preventive responses. The review also addresses the requirements for moving beyond benchmark performance toward interpretable, robust, equitable, externally and prospectively validated AI systems capable of improving real-world clinical decisions (Kelly et al., 2019; Sendak et al., 2020; Vollmer et al., 2020; Ghassemi et al., 2021; Vasey et al., 2022).

## 2 Review methodology

This article was designed as a structured narrative review informed by targeted literature searches. Relevant publications were identified through PubMed/MEDLINE, Scopus, and Web of Science Core Collection up to May 2026, without a predefined lower date limit, to include both foundational and recent evidence. The searches were conducted by two authors, and retrieved references were imported into EndNote for reference management and duplicate removal. Particular attention was given to studies published from 2019 onward because of the rapid development of artificial intelligence (AI), digital and blood-based biomarkers, and translational machine-learning applications.

Search terms combined “cognitive decline,” “mild cognitive impairment,” “dementia,” “Alzheimer’s disease,” and “neurodegenerative diseases” with “artificial intelligence,” “machine learning,” “deep learning,” “multimodal biomarkers,” “digital phenotyping,” “risk prediction,” “patient stratification,” “lifestyle intervention,” “multidomain prevention,” “natural medicines,” “natural products,” “nutraceuticals,” “clinical translation,” and “prospective validation.” Terms were adapted to the syntax of each database.

Eligible publications included peer-reviewed original studies, randomized controlled trials, cohort studies, diagnostic and prognostic investigations, systematic reviews, meta-analyses, clinical guidelines, consensus statements, and relevant methodological papers. Studies were included when they addressed cognitive decline or major neurodegenerative disorders and provided evidence on AI- or ML-based prediction, multimodal data integration, biomarkers, digital phenotyping, prevention-oriented interventions, or natural-compound prioritization. Studies unrelated to cognitive decline, lacking sufficient methodological detail, available only as abstracts or non-peer-reviewed commentary, or focused exclusively on technical performance without clinical or preventive relevance were excluded. After duplicate removal, titles and abstracts were screened independently by two authors. Disagreements regarding potential relevance were resolved through discussion and consensus. Articles considered potentially eligible by either reviewer were retained for full-text assessment, which was performed by a senior author against the predefined inclusion and exclusion criteria.

Reference lists of key reviews, guidelines, and landmark studies were also screened. Because of the interdisciplinary and narrative nature of the review, no formal meta-analysis or risk-of-bias assessment was performed. As the review was conceived as a structured narrative synthesis rather than a systematic review, study selection was not documented through a prospective numerical record or a PRISMA flow diagram. Evidence was synthesized thematically to develop an AI-enabled framework linking multimodal risk assessment, precision prevention, intervention personalization, and clinical implementation.

## 3 Neurodegenerative diseases as a multimodal challenge for AI-enabled prevention

### 3.1 Clinical and biological heterogeneity of cognitive decline

The DSM-5 classifies dementia within Major Neurocognitive Disorders and recognizes impairment across memory, language, executive function, attention, social cognition, and visuospatial abilities (American Psychiatric Association, 2013). However, syndromic classification must be integrated with disease-specific criteria because neurodegenerative disorders differ substantially in clinical presentation, temporal evolution, and underlying biology (McKhann et al., 2011; Postuma et al., 2015; McKeith et al., 2017).

Cognitive decline may emerge through different combinations of amnestic, executive, linguistic, behavioral, psychiatric, visuospatial, or motor alterations. Some disorders initially present with motor, behavioral, psychiatric, or language symptoms, with cognitive impairment appearing later in the disease course (McKeith et al., 2017; Rascovsky et al., 2011; McColgan and Tabrizi, 2018). Alzheimer’s disease, vascular cognitive impairment, Lewy body disorders, frontotemporal dementia, Huntington’s disease, and mixed dementias consequently show partially distinct but overlapping clinical, pathological, imaging, and biomarker profiles (McKhann et al., 2011; Postuma et al., 2015; McKeith et al., 2017; Rascovsky et al., 2011; McColgan and Tabrizi, 2018; Skrobot et al., 2018; Schneider et al., 2007; Jellinger and Attems, 2010). Mixed neurodegenerative and cerebrovascular pathologies are particularly frequent in older populations, limiting rigid single-disease classifications and single-marker approaches.

This clinicobiological heterogeneity provides a central rationale for AI-enabled multimodal modeling. However, multimodal integration should remain disease-specific, because the clinically relevant features, biomarker hierarchies, progression trajectories, and prediction targets differ substantially across disorders. Integrating cognitive, behavioral, neuroimaging, fluid-biomarker, genetic, vascular, and longitudinal data may support more refined phenotyping, differential diagnosis, and individualized prediction than models based on isolated clinical scores or biomarkers.

### 3.2 Shared pathophysiological mechanisms as multimodal targets for AI

Despite their clinical heterogeneity, neurodegenerative diseases share interconnected pathological processes that may develop during prolonged preclinical phases. These include protein misfolding and aggregation—such as amyloid-β and tau in Alzheimer’s disease, α-synuclein in Parkinson’s disease and dementia with Lewy bodies, mutant huntingtin in Huntington’s disease, and TDP-43 or SOD1 in ALS/FTD-related disorders—together with impaired proteostasis, mitochondrial dysfunction, oxidative stress, neuroinflammation, and synaptic failure (Hardy and Higgins, 1992; Long and Holtzman, 2019; Soto and Pritzkow, 2018; Rossi et al., 2023).

Mitochondrial and metabolic dysfunction can amplify oxidative injury through impaired energy production, calcium regulation, mitochondrial dynamics, and mitophagy, while maladaptive activation of microglia and astrocytes may exacerbate proteinopathy, synaptic disruption, and neuronal vulnerability (Heneka et al., 2015; Patani et al., 2023; Adamu et al., 2024; Peggion et al., 2024; Mayer et al., 2024). Synaptic dysfunction and loss may arise before extensive neuronal death, providing potentially measurable signals for earlier detection and intervention (Camporesi et al., 2020; Nilsson et al., 2024).

Because these mechanisms interact across molecular, cellular, and neural-network levels, isolated biomarkers may incompletely represent disease stage or trajectory. AI-based multimodal models may integrate proteostatic, metabolic, inflammatory, synaptic, imaging, and clinical indicators to identify composite signatures associated with progression, treatment response, and precision-prevention opportunities.

### 3.3 Genetic, environmental, and lifestyle determinants across the life course

Neurodegenerative diseases are increasingly understood as disorders of declining brain resilience shaped by interactions between genetic susceptibility and cumulative exposures across the life course. Highly penetrant mutations account for only a small proportion of cases, whereas common late-onset disorders generally have complex and probabilistic genetic architectures. APOE ε4 is the strongest common genetic risk factor for sporadic Alzheimer’s disease, while GBA1 and LRRK2 variants contribute substantially to Parkinson’s disease susceptibility (Serrano-Pozo et al., 2021; Smith et al., 2022).

In most sporadic neurodegenerative diseases, genetic vulnerability interacts with vascular and metabolic burden, diet, physical inactivity, sleep disruption, psychosocial adversity, reduced cognitive and social engagement, and environmental pollutants. These exposures may influence biological aging and age at symptom onset through gene–environment interactions and epigenetic regulation (Livingston et al., 2024; Migliore and Coppedè, 2022; Shi et al., 2018; Li et al., 2024). Rather than operating independently, they converge on vascular injury, metabolic dysregulation, chronic inflammation, and reduced cognitive reserve, progressively weakening brain resilience (Livingston et al., 2024).

Their interdependence, temporal variability, and heterogeneous effects limit static and single-factor risk models. AI-enabled approaches may integrate these determinants into multidimensional and temporally dynamic risk profiles. Their use for selecting and adapting preventive interventions is discussed in Section 4.

### 3.4 Multimodal biomarkers and AI-enabled preclinical detection

Within the biologically informed framework introduced above, amyloid, tau, and neurodegeneration biomarkers provide complementary information on pathological state and disease progression. Longitudinal studies indicate that amyloid deposition may begin 15–20 years before dementia, followed by tau propagation, synaptic dysfunction, and progressive neurodegeneration, thereby creating a potential window for preventive and disease-modifying intervention (Bateman et al., 2012; Villemagne et al., 2013).

Blood-based biomarkers provide increasingly scalable tools for preclinical detection and risk stratification. Plasma p-tau217 and p-tau231 show high diagnostic accuracy for Alzheimer’s disease, while the amyloid-β42/40 ratio, neurofilament light chain, and glial fibrillary acidic protein provide complementary information on amyloid pathology, neuroaxonal injury, progression, and astroglial activation (Janelidze et al., 2021; Ashton et al., 2022; Nakamura et al., 2018; Schindler et al., 2019; Disanto et al., 2017; Verberk et al., 2021; Pereira et al., 2021). Multimarker panels may therefore support screening, prognosis, trial enrichment, and treatment-response monitoring (Teunissen et al., 2022; Hampel et al., 2023; Grande et al., 2025).

Neuroimaging provides complementary molecular, structural, functional, and vascular information. Tau PET enables *in vivo* staging of neurofibrillary pathology, while functional-connectivity and diffusion MRI may reveal network and white-matter alterations before overt atrophy or clinical decline (Leuzy et al., 2020; Ossenkoppele et al., 2022; Badhwar et al., 2017). Retinal imaging has also been investigated as a non-invasive indicator of cerebral amyloid and vascular pathology, although its clinical role remains under evaluation (den Haan et al., 2018; Hadoux et al., 2019).

Digital biomarkers derived from speech, gait, sleep, device interaction, actigraphy, smartphones, and wearable sensors may complement episodic clinical assessments by capturing continuous within-person changes in real-world settings (Insel, 2017; Kourtis et al., 2019; Lott et al., 2024).

The integration of fluid, imaging, cognitive, genetic, clinical, and digital measures provide a strong rationale for AI- and ML-based preclinical detection. Multimodal models may identify composite signatures, support biological staging and individualized risk estimation, and track longitudinal change more effectively than isolated biomarkers. However, clinical implementation requires assay and acquisition harmonization, management of missing or asynchronous data, representative cohorts, external and prospective validation, calibration, interpretability, and ethical management of preclinical risk disclosure. Their value should therefore be judged by their capacity to improve preventive decision-making, not by discrimination performance alone.

### 3.5 Mild cognitive impairment as a target for AI-enabled risk prediction and early intervention

Mild cognitive impairment (MCI) is a heterogeneous syndrome characterized by objective cognitive decline with substantially preserved functional independence. It includes amnestic and non-amnestic presentations arising from different underlying pathologies, while biomarker-positive cases may be classified within the symptomatic pre-dementia stages of the Alzheimer’s disease continuum (Petersen et al., 2014; Jack et al., 2018, 2024).

Prognosis is highly variable: some individuals progress to dementia, whereas others remain stable or return to cognitively unimpaired status. Trajectories are influenced by pathological burden, cognitive reserve, cerebrovascular disease, and lifestyle-related factors, making MCI a probabilistic risk state rather than an invariably progressive condition (Mitchell and Shiri-Feshki, 2009; Canevelli et al., 2016; Kapasi et al., 2017; Dichgans and Leys, 2017). Biomarker profiles combining amyloid, tau, neurodegeneration, astroglial activation, and neuronal injury may improve prognostic assessment, although mixed pathologies limit reliance on any single marker (Vos et al., 2015; Hampel et al., 2023; Pereira et al., 2021).

Cognitive composites, digital testing, speech analysis, neuroimaging, and smartphone-based monitoring provide complementary measures of subtle and longitudinal change (Donohue et al., 2014; Jack et al., 2010; Badhwar et al., 2017; Kourtis et al., 2019). AI- and ML-based models may integrate these data with vascular, genetic, clinical, and lifestyle information to distinguish relatively stable individuals from those at greater risk of progression, define prediction horizons, identify clinically meaningful subgroups, and support trial enrichment and personalized monitoring.

The clinical relevance of this stratification has increased with disease-modifying therapies for early Alzheimer’s disease. Trials of lecanemab and donanemab included patients with MCI or mild dementia and biomarker-confirmed amyloid pathology, demonstrating modest slowing of decline together with important safety-monitoring requirements (van Dyck et al., 2023; Sims et al., 2023). MCI therefore represents a key stage for AI-enabled precision prevention, provided that multimodal predictions are prospectively validated and translated into clinically actionable estimates of progression, treatment eligibility, expected benefit, and risk.

## 4 AI-enabled precision prevention: modifiable risk, multidomain interventions, and environmental determinants

### 4.1 Modifiable risk factors as inputs for personalized prevention

Dementia risk is increasingly understood as the cumulative result of modifiable and non-modifiable determinants acting from early life to older age. The 2024 Lancet Commission estimated that addressing 14 factors—including educational disadvantage, sensory impairment, vascular and metabolic conditions, behavioral exposures, psychosocial factors, traumatic brain injury, and air pollution—could theoretically prevent or delay up to 45% of dementia cases (Livingston et al., 2024; Liang Z. et al., 2021; Patnode et al., 2020; Lee et al., 2025).

These determinants vary in relevance across the life course and frequently cluster rather than act independently. Educational attainment may influence cognitive reserve, whereas vascular, metabolic, sensory, behavioral, and social factors may contribute to later risk through chronic inflammation, oxidative stress, endothelial dysfunction, impaired cerebral perfusion, and reduced neural resilience. Evidence particularly supports the importance of smoking cessation, avoidance of harmful alcohol consumption, regular physical activity, cardiometabolic control, and adherence to Mediterranean-type, DASH, or MIND dietary patterns (Howard et al., 1998; Deal et al., 2020; Sabia et al., 2018; Zhang et al., 2026; Scarmeas et al., 2006; Féart et al., 2009; Lourida et al., 2013; Liu et al., 2018; Johnston et al., 2025; Chen and Nakagawa, 2023; Iso-Markku et al., 2024). Overall, prevention is likely to depend on sustained combinations of measures rather than isolated interventions introduced late in life (Dhana et al., 2020; Lourida et al., 2019).

However, population-level associations cannot determine which preventive targets should be prioritized for an individual or how expected benefit may vary according to age, sex, genetics, cognition, comorbidities, socioeconomic context, exposure history, and adherence. AI- and ML-based models may integrate these variables to estimate individualized risk, identify interacting exposure patterns, prioritize modifiable targets, and update recommendations as biological, clinical, and behavioral profiles change over time. Their value lies in translating multidomain evidence into dynamic prevention strategies rather than merely reproducing conventional population-level risk scores.

**FIGURE 2:**
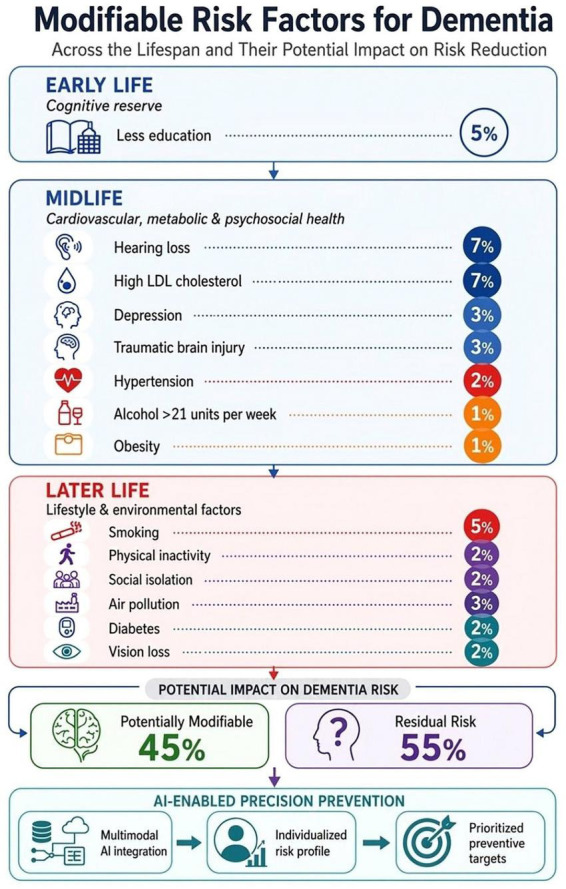
Life-course distribution of the 14 modifiable dementia risk factors identified by the 2024 Lancet Commission and their relevance to AI-enabled precision prevention. Early-life factors primarily influence cognitive reserve, whereas mid- and later-life risk reflects cardiovascular, metabolic, sensory, behavioral, psychosocial, and environmental determinants. Together, these factors account for an estimated 45% of potentially modifiable dementia risk. Their integration with clinical and biological data may support individualized risk estimation and prioritization of preventive targets. Created in BioRender.com.

The life-course distribution of modifiable dementia risk factors and their potential contribution to risk reduction are summarized in Figure 2.

### 4.2 Lifestyle and cardiovascular targets for AI-enabled intervention personalization

Physical activity, cardioprotective dietary patterns, cognitive enrichment, and cardiovascular-risk control represent complementary targets for dementia prevention and may influence cerebral perfusion, metabolic function, inflammation, oxidative balance, neuroplasticity, and cognitive reserve (Gorelick et al., 2011; Deckers et al., 2015; World Health Organization, 2019; Livingston et al., 2024).

Regular physical activity is associated with more favorable late-life cognitive outcomes, although intervention effects are generally modest and heterogeneous (Pedersen, 2019; Lourenco et al., 2019; Severinsen and Pedersen, 2020; Chow et al., 2022; Lee et al., 2021; Iso-Markku et al., 2024). Adherence to Mediterranean-style and MIND dietary patterns has similarly been associated with lower dementia risk and better cognitive trajectories, while PREDIMED-NAVARRA reported cognitive benefits from a Mediterranean diet supplemented with extra-virgin olive oil or nuts (Scarmeas et al., 2006; Féart et al., 2009; Lourida et al., 2013; Martínez-Lapiscina et al., 2013; Estruch et al., 2018; Chen et al., 2023; Nucci et al., 2024).

Cognitive reserve may modify the clinical expression of neuropathological burden, whereas management of hypertension, diabetes, obesity, and dyslipidemia may reduce vascular contributions to cognitive impairment (Gorelick et al., 2011; Wolters et al., 2018; Liu et al., 2024a,b; Lee et al., 2025). In SPRINT MIND, intensive systolic blood-pressure control reduced the incidence of mild cognitive impairment, although the effect on probable dementia was not statistically significant (Williamson et al., 2019).

The effectiveness of these strategies varies according to baseline risk, comorbidities, adherence, intervention intensity, and individual biological and behavioral profiles (Livingston et al., 2024). AI and ML may integrate lifestyle, cardiovascular, clinical, biomarker, and longitudinal data to prioritize preventive targets and adapt recommendations over time. Wearables, mobile platforms, sleep and mobility measures, speech analysis, and remote cognitive assessment may additionally support adherence and response monitoring (Kourtis et al., 2019; Kivipelto et al., 2020; Lott et al., 2024). However, AI-supported recommendations require prospective validation and should remain interpretable, equitable, feasible, and subordinate to clinical judgment and person-centered care (World Health Organization, 2021; Ghassemi et al., 2021; Vasey et al., 2022).

### 4.3 Multidomain prevention: evidence from intervention studies

Multidomain prevention programmes combine dietary counseling, physical activity, cognitive training, and vascular and metabolic risk management to address the interacting determinants of cognitive decline (Ngandu et al., 2015; Livingston et al., 2024). The FINGER trial provided proof of concept by demonstrating that a 2-year intervention improved or maintained cognitive performance in at-risk older adults compared with general health advice (Ngandu et al., 2015).

However, subsequent trials produced heterogeneous findings. MAPT did not show a significant benefit on its primary cognitive outcome, while preDIVA did not reduce all-cause dementia incidence in the overall population (Andrieu et al., 2017; Moll van Charante et al., 2016). A systematic review and meta-analysis nevertheless identified small but significant benefits for cognitive composite outcomes and dementia-related risk measures, although effects on global cognition were inconsistent (Meng et al., 2022). These findings suggest that effectiveness depends on baseline risk, intervention intensity and duration, adherence, comorbidities, cognitive reserve, and the quality of usual care.

This response heterogeneity provides a rationale for AI- and ML-supported personalization. Models integrating clinical characteristics, modifiable risks, biomarkers, cognition, adherence, and longitudinal digital measures may help identify likely responders, prioritize intervention components, and adapt their intensity over time. Wearables, mobile platforms, and remote cognitive assessments may additionally support monitoring of adherence and response (Kourtis et al., 2019; Lott et al., 2024).

The World-Wide FINGERS network is adapting FINGER-based programmes across countries and healthcare settings, providing an important context for population-specific stratification and implementation research (Kivipelto et al., 2020). The clinical value of AI-guided personalization will require prospective validation and evidence that it improves adherence, cognitive outcomes, or biomarker trajectories beyond standard multidomain interventions.

### 4.4 Environmental exposures as inputs for AI-enabled risk modeling

Environmental exposures may contribute to neurodegenerative risk through inflammatory, oxidative, vascular, and neurovascular pathways. Outdoor air pollution currently has the strongest epidemiological support: the 2024 Lancet Commission recognizes it as a modifiable dementia risk factor, while meta-analyses associate long-term exposure to PM2.5, nitrogen dioxide, and black carbon with incident dementia (Livingston et al., 2024; Best Rogowski et al., 2025; Huang et al., 2025).

Micro- and nanoplastics (MNPs) represent a less characterized candidate exposure. Polymeric particles have been detected in blood, cerebrospinal fluid, and post-mortem brain tissue, with preliminary studies reporting higher burdens in dementia cases or amyloid-positive individuals (Nihart et al., 2025; He et al., 2025; Bu et al., 2026). Preclinical evidence suggests possible associations with oxidative stress, neuroinflammation, mitochondrial dysfunction, blood–brain barrier disruption, and cognitive impairment (Skaba et al., 2025; Zheng et al., 2024; Araújo et al., 2025). However, current human evidence remains predominantly cross-sectional and associative, with interpretation limited by small samples, heterogeneous analytical procedures, uncertain exposure assessment, and insufficient longitudinal data (Gecegelen et al., 2025; Araújo et al., 2025).

Within AI-enabled precision prevention, environmental variables may complement genetic, clinical, socioeconomic, lifestyle, and biomarker data by contributing information on cumulative and geographically patterned exposures. ML models may help identify non-linear interactions and population subgroups in whom environmental determinants contribute disproportionately to cognitive risk. Their reliability will nevertheless depend on standardized measurements, representative longitudinal datasets, transparent management of missing data, and external validation. Air pollution may currently be treated as an established population-level determinant, whereas MNPs should remain an exploratory research variable rather than a clinically actionable predictor (Best Rogowski et al., 2025; Huang et al., 2025; Gecegelen et al., 2025; Araújo et al., 2025).

Environmental exposure should therefore be considered a contextual layer complementing, rather than redefining, validated biomarker frameworks. The proposed extension from ATN to an “ATNE” framework remains conceptual and is more appropriately situated within the exposome perspective, which considers interactions between lifelong exposures, genetic susceptibility, and biological vulnerability (Finch and Kulminski, 2019). Environmental variables may eventually contribute to individualized risk estimation, but current evidence does not support their use as a formal diagnostic category, routine screening measure, or basis for treatment selection.

## 5 Natural medicines and nutraceuticals in AI-enabled precision prevention

Natural medicines and nutraceuticals represent chemically complex and biologically pleiotropic sources of candidate interventions for cognitive decline. Their chemical diversity, multitarget activity, variable composition, and incomplete pharmacological characterization make conventional candidate selection and clinical translation particularly challenging. However, their translation into precision prevention depends on reproducible chemical characterization, bioavailability and brain exposure, target engagement, patient stratification, and measurable cognitive or biomarker effects (Cui et al., 2023; Goyal et al., 2024; Nahar et al., 2025). Within this context, the relevance of natural medicines to the present review lies primarily in their role as a complex use case for AI-enabled integration and prioritization. AI and machine learning may support this pathway by integrating phytochemical, pharmacological, omics, biomarker, and clinical data to prioritize candidates, identify potential responder profiles, optimize formulations, and support biomarker-informed clinical development (Mullowney et al., 2023; Hampel et al., 2023; Zhang J. et al., 2025; Zhang K. et al., 2025).

### 5.1 Neuroprotective candidates and current clinical evidence

Natural compounds of plant, fungal, microalgal, and marine origin provide structurally diverse candidates with multitarget biological activity. These candidates span diverse chemical classes, including polyphenols, terpenoids, alkaloids, carotenoids, fatty acids, and polysaccharides (Cui et al., 2023; Hu et al., 2023; Bonetto et al., 2025; Nahar et al., 2025). Although numerous individual compounds have shown antioxidant, anti-inflammatory, neuroprotective, or cognitive effects in experimental studies, the breadth and heterogeneity of this evidence make conventional prioritization difficult. Most of these findings derive from preclinical models and therefore support biological plausibility and candidate prioritization rather than clinical efficacy. This complexity provides a rationale for AI-assisted comparison of chemical structure, biological activity, pharmacokinetics, target profiles, and translational evidence across candidate compounds (Wiciñski et al., 2020; Costa et al., 2016; Bhat et al., 2022; Küpeli Akkol et al., 2022; Wang W. et al., 2024; Wei et al., 2023; Welty, 2023; Adıgüzel and Ülger, 2024; Dan et al., 2023).

For primary prevention, current evidence more strongly supports sustained dietary patterns than isolated compounds. Mediterranean- and MIND-type dietary patterns, including diets rich in flavonoids, extra-virgin olive oil, and nuts, have been associated with lower dementia risk or more favorable cognitive outcomes, although the magnitude of benefit varies across populations and baseline-risk profiles (Martínez-Lapiscina et al., 2013; Morris et al., 2015; Valls-Pedret et al., 2015; Jennings et al., 2024). However, much of this evidence is observational and cannot establish causality because residual confounding, socioeconomic and lifestyle differences, and reverse causation may influence the reported associations. Randomized dietary-intervention studies provide stronger evidence, but remain limited in number and show heterogeneous effects across populations, intervention designs, and cognitive endpoints. Evidence for individual bioactives, including carotenoids, remains less consistent and is insufficient to establish a preventive effect on dementia incident (Davinelli et al., 2021). AI-based integration of dietary exposure, vascular risk, genetic susceptibility, biomarkers, and longitudinal cognitive data may help identify subgroups more likely to benefit from specific dietary or nutraceutical strategies and distinguish population-level associations from individualized preventive effects.

Evidence for secondary prevention and adjunctive treatment is less consistent and derives mainly from mild cognitive impairment, early Alzheimer’s disease, and vascular brain-injury populations. Selected interventions, including polyphenol-based preparations, resveratrol, huperzine A, omega-3 fatty acids, standardized botanical extracts, and microbiota-targeted approaches, have shown signals of cognitive, functional, or biomarker benefit in specific clinical settings (Turner et al., 2015; Yang et al., 2013; Chatzikostopoulos et al., 2024). However, randomized clinical evidence remains limited and is frequently based on small or exploratory trials. Positive findings should therefore be considered provisional unless replicated in adequately powered studies using standardized preparations, appropriate comparators, sufficient treatment duration, and clinically meaningful cognitive or functional endpoints. In addition, treatment effects are generally modest and appear to vary according to disease stage, formulation, dose, treatment duration, endpoint selection, vascular burden, and genetic background, as illustrated by differential responses observed in APOE ε4 carriers (Shinto et al., 2024). This marked heterogeneity limits direct comparison across studies but provides a rationale for AI-assisted integration of clinical, biomarker, pharmacokinetic, genetic, and treatment-related variables to identify potential responder profiles and more informative outcome patterns (Small et al., 2018; Buglio et al., 2022; Morató et al., 2023; Brickman et al., 2014; Gratton et al., 2020; Baek et al., 2024; Aljumaah et al., 2022; Haskell-Ramsay et al., 2022; Ni Lochlainn et al., 2024).

Natural bioactives should therefore be regarded as supportive preventive or adjunctive strategies rather than established stand-alone disease-modifying interventions. Overall, the current evidence hierarchy supports mechanistic plausibility and candidate prioritization at the preclinical level, hypothesis generation at the observational level, and only provisional clinical applicability for most individual natural medicines because robust and replicated randomized evidence remains limited. Their broad chemical diversity, variable composition, incomplete target annotation, and heterogeneous evidence make conventional candidate selection and translation inefficient and poorly reproducible. AI- and ML-based integration of chemical structures, phytochemical fingerprints, bioactivity profiles, omics data, pharmacokinetics, biomarkers, and clinical characteristics may help prioritize candidates, rank mechanistically coherent preparations, and identify subgroups more likely to respond. The principal contribution of AI in this context is therefore not to infer efficacy from mechanistic complexity, but to reduce the candidate space and generate testable, biomarker-informed hypotheses for experimental and clinical validation. Nevertheless, computational prioritization cannot replace confirmation of chemical identity, reproducible bioactivity, brain exposure, safety, target engagement, and clinically meaningful effects (Mullowney et al., 2023; Othman et al., 2025; Durant et al., 2024).

### 5.2 Mechanistic domains of neuroprotection

Natural compounds may influence multiple interconnected mechanisms implicated in cognitive decline, including oxidative stress, mitochondrial dysfunction, neuroinflammation, protein aggregation, impaired autophagy, blood–brain barrier disruption, cerebrovascular injury, synaptic dysfunction, and microbiota-related metabolic alterations. Rather than representing independent lines of evidence, these mechanisms constitute interconnected biological domains that may be jointly modeled through AI- and ML-based integration of molecular, omics, imaging, biomarker, and phenotypic data. Clinical evidence nevertheless remains substantially more limited than preclinical evidence, and computational or mechanistic convergence should not be interpreted as proof of clinical efficacy (Cui et al., 2023; Nahar et al., 2025).

#### 5.2.1 Oxidative, mitochondrial, and neuroimmune mechanisms

Oxidative stress, impaired mitochondrial function, and sustained neuroimmune activation contribute to synaptic dysfunction, neuronal injury, and neurodegenerative progression (Misrani et al., 2021). Selected clinical studies of standardized botanical preparations and polyphenolic compounds have reported changes in oxidative-stress, inflammatory, or neurodegeneration-related biomarkers in mild cognitive impairment or Alzheimer’s disease, but the findings remain limited, heterogeneous, and difficult to compare across products and populations (Buglio et al., 2022; Morató et al., 2023; Liu et al., 2025). This variability highlights the need for AI-assisted integration of biomarker patterns, treatment characteristics, and patient-level features rather than interpretation based on isolated molecular endpoints.

Preclinical studies indicate that diverse natural-product classes may modulate interconnected oxidative, mitochondrial, inflammatory, and cell-death processes through partially overlapping signaling networks. However, the multiplicity of compounds, assays, pathways, and experimental models makes it difficult to determine which effects are specific, reproducible, and translationally relevant (Yang et al., 2009, 2023; Khan et al., 2018; Grimmig et al., 2018; Zhang et al., 2017; Yu et al., 2018a; Taksima et al., 2019; Park et al., 2020; Zhu et al., 2020; Ahmad et al., 2021; Cong et al., 2021; Wu et al., 2021; Xiang et al., 2021; Duan et al., 2022; Ho et al., 2022; Kou et al., 2022; Qiao et al., 2022; He et al., 2023; Jiang Y. et al., 2023; Vongthip et al., 2024; Fang et al., 2025). AI- and ML-based approaches may help compare these multidimensional profiles and identify convergent signatures associated with stronger mechanistic coherence and higher translational potential.

AI- and ML-based integration of chemical, transcriptomic, proteomic, cytokine, mitochondrial, and phenotypic data may help distinguish non-specific antioxidant or anti-inflammatory effects from compound-specific mechanistic signatures, rank candidates according to the consistency of their multimodal profiles, and identify candidate responder profiles (Mullowney et al., 2023; Zhang J. et al., 2025; Zhang K. et al., 2025). However, computationally identified signatures remain hypothesis-generating, and translation requires adequate brain exposure, central target engagement, reproducible biomarker modulation, and clinically meaningful cognitive effects.

#### 5.2.2 Proteostasis, neurovascular integrity, and synaptic function

Impaired proteostasis, protein aggregation, defective autophagy–lysosomal clearance, blood–brain barrier dysfunction, cerebrovascular injury, and synaptic failure represent interconnected mechanisms of neurodegeneration. Selected clinical studies of natural-product-based interventions have reported changes in amyloid- or tau-related biomarkers, vascular and metabolic measures, cognition, or activities of daily living. However, these findings remain heterogeneous across preparations, populations, and endpoints, and direct evidence of disease modification is limited (Turner et al., 2015; Yang et al., 2013; Brickman et al., 2014; Neshatdoust et al., 2016; Small et al., 2018; Barnes et al., 2021; Gratton et al., 2020; Baek et al., 2024; Carrillo et al., 2025). AI-assisted integration of pathological, vascular, synaptic, pharmacokinetic, and clinical data may help determine whether apparently distinct effects converge into reproducible and clinically relevant response profiles.

Preclinical studies suggest that diverse natural compounds may modulate interconnected processes involving protein aggregation and clearance, autophagy, neurovascular integrity, synaptic maintenance, neurotransmission, and neurotrophic signaling. However, the large number of compounds, targets, pathways, and experimental models makes mechanistic comparison and candidate prioritization difficult (Bieschke et al., 2010; Sadegh Malvajerd et al., 2019; Lee et al., 2020; Wicha et al., 2020; Ahmad et al., 2021; Xiong et al., 2021; Liang Y. et al., 2021; Zhang et al., 2022; Suzuki et al., 2022; Liu et al., 2024c,d; Hu et al., 2024; Jiang N. et al., 2023; Wang et al., 2023; Sun et al., 2025; Ali and Datusalia, 2025). AI- and ML-based models may help identify compounds or preparations showing convergent effects across pathological, vascular, and synaptic domains rather than isolated activity in a single assay or pathway.

Multimodal AI models may integrate structural, biochemical, imaging, vascular, electrophysiological, transcriptomic, proteomic, and behavioral data to identify compounds with convergent effects on aggregation, neurovascular integrity, and synaptic function. They may also support candidate ranking by distinguishing broad, non-specific activity from reproducible multimodal signatures associated with greater translational relevance. However, these outputs remain hypothesis-generating and require orthogonal confirmation of compound identity, mechanism, brain exposure, pathological-protein modulation, barrier-related effects, and clinically meaningful cognitive benefit (Mullowney et al., 2023; Zhang J. et al., 2025; Zhang K. et al., 2025).

#### 5.2.3 Gut-microbiota-brain axis and metabolic regulation

The gut-microbiota-brain axis links microbial composition and metabolism with intestinal-barrier integrity, immune regulation, mitochondrial function, neuroinflammation, and cognition. Selected clinical studies of probiotic, prebiotic, and diet-based interventions have reported microbiome changes accompanied by cognitive or metabolic signals of benefit. However, the evidence remains preliminary and heterogeneous, and causal relationships between microbial changes and cognitive outcomes have not been established (Aljumaah et al., 2022; Haskell-Ramsay et al., 2022; Ni Lochlainn et al., 2024). AI-assisted integration of microbial, metabolomic, dietary, clinical, and cognitive data may help distinguish reproducible response patterns from background interindividual variability.

Preclinical evidence indicates that diverse natural compounds may influence cognitive or cerebrovascular outcomes through microbiota remodeling, modulation of microbial metabolites, preservation of gut-barrier integrity, regulation of neuroinflammation, and effects on mitochondrial and amyloid-related processes (Liu et al., 2021; Zhang et al., 2023; Duan et al., 2023; Lamichhane et al., 2024; Sun et al., 2024; Xu et al., 2024; Altendorfer et al., 2025; Wang et al., 2026). These effects span multiple biological levels and are strongly influenced by host, dietary, microbial, and experimental variables, making direct comparison across studies difficult (Zhang B. et al., 2025). AI- and ML-based analysis may help link specific microbial and metabolic changes with downstream inflammatory, mitochondrial, and cognitive outcomes, thereby identifying mechanistically coherent candidate profiles.

Because microbiome and metabolomic data are high-dimensional, dynamic, and strongly influenced by diet, medication, geography, and host characteristics, they are particularly suited to AI- and ML-based analysis. Computational models may identify microbial and metabolic signatures, predict bioactive–microbiota interactions, link treatment-induced microbial changes with downstream biological and cognitive outcomes, and identify potential responder profiles. Their value will depend on whether these models can generate reproducible and clinically interpretable predictions rather than merely classify complex datasets. Clinical translation will nevertheless require standardized sampling, sequencing, dietary assessment, confounder control, external validation, and prospective demonstration that microbiome changes are associated with reproducible cognitive or biomarker benefits (Mullowney et al., 2023; Zhang J. et al., 2025; Zhang K. et al., 2025).

### 5.3 AI-assisted discovery, screening, prioritization, and formulation

The discovery and development of neuroprotective natural compounds are complicated by broad chemical diversity, pleiotropic activity, incomplete target annotation, mixture complexity, variable provenance, and heterogeneous experimental evidence. AI and ML may integrate molecular structures, phytochemical and spectral fingerprints, bioactivity profiles, omics data, pharmacological information, and literature-derived rank candidates, identify mechanistically coherent compound–target relationships, and reduce reliance on conventional trial-and-error screening (Mullowney et al., 2023; Zhang J. et al., 2025; Zhang K. et al., 2025). In this setting, the principal value of AI lies in filtering heterogeneous evidence and prioritizing experimentally testable candidates rather than replacing chemical or biological validation.

Relevant applications include bioactivity prediction, compound–target interaction inference, multitarget profiling, virtual screening, scaffold prioritization, toxicity assessment, pharmacokinetic prediction, blood–brain barrier permeability estimation, and synthetic-feasibility analysis. These tasks may be addressed through graph-based models, chemical language models, descriptor-based approaches, and generative architectures. However, model performance depends strongly on dataset size and quality, annotation reliability, chemical-space coverage, validation strategy, and interpretability (Nguyen et al., 2021; Liu et al., 2022; Sultan et al., 2024; Grant et al., 2025; Ekins and Lane, 2025). For central nervous system applications, integrated pipelines combining predicted activity with ADMET, toxicity, pharmacokinetic, and BBB-permeability filters are more informative than isolated ranking models (Grant et al., 2025; Zhang J. et al., 2025). Such multistage pipelines may reduce false-positive prioritization by excluding candidates that appear biologically active but lack adequate safety, exposure, or central nervous system accessibility.

Open natural-product resources provide important data infrastructures for these workflows. The Global Natural Products Social Molecular Networking platform supports tandem mass-spectrometry data sharing, molecular networking, annotation, and dereplication in complex extracts (Wang et al., 2016). The Natural Product Activity and Species Source database links compound structures with biological activities and source organisms, while COCONUT aggregates open and provenance-aware natural-product structures and metadata for curation and computational screening (Zeng et al., 2018; Zhao et al., 2023; Sorokina et al., 2021; Chandrasekhar et al., 2025). Together, these resources support annotation, activity prediction, candidate ranking, and prioritization for experimental validation (Zeng et al., 2024). Their value for AI-assisted discovery depends, however, on data curation, standardized chemical identifiers, provenance tracking, harmonized activity annotations, and adequate representation of the chemical diversity present in natural products.

Generative models may further propose natural-product-inspired analogues or scaffold modifications intended to improve potency, selectivity, solubility, metabolic stability, or drug-like properties (Shen et al., 2024; Das, 2025; Liu et al., 2025). When combined with activity, ADMET, toxicity, and blood–brain barrier constraints, these models may help prioritize chemically plausible candidates for experimental testing. However, computationally generated candidates may remain unsuitable because of limited synthetic accessibility, toxicity, instability, inadequate brain exposure, or poor biological efficacy. AI-generated rankings and structures should therefore be considered hypothesis-generating outputs that require chemical synthesis, analytical confirmation, and biological validation before any therapeutic inference can be made.

Formulation development represents a complementary translational application. Many natural compounds show poor aqueous solubility, low oral bioavailability, rapid metabolism, chemical instability, and insufficient systemic or central nervous system exposure (Goyal et al., 2024; Ureña-Vacas et al., 2024). Liposomes, polymeric nanoparticles, and other delivery systems may improve stability, circulation time, and brain-directed exposure, although most supporting evidence remains preclinical and unresolved issues include scale-up, biodistribution, safety, reproducibility, and regulatory complexity (Huang et al., 2024; Kamath et al., 2024; Sadat Razavi et al., 2025). AI and ML may assist formulation optimization by integrating molecular descriptors, carrier properties, release profiles, biodistribution, and biological outcomes to predict formulation performance, identify critical formulation variables, and prioritize compound–delivery system combinations with more favorable exposure and safety profiles (Ekins and Lane, 2025; Othman et al., 2025). However, such models require standardized formulation datasets and prospective experimental validation, because predictions derived from small or heterogeneous preclinical datasets may not generalize across compounds, carriers, or biological models.

Across discovery and formulation, model outputs require orthogonal chemical and biological validation. Candidate compounds and formulations must undergo confirmation of identity, purity, reproducible bioactivity, mechanism, pharmacokinetics, brain penetration, safety, target engagement, and clinically meaningful effects. The reliability of AI-supported development ultimately depends on representative datasets, standardized characterization, external validation, and experimentally constrained workflows rather than on autonomous end-to-end prediction or model performance assessed only within benchmark datasets (Durant et al., 2024; Othman et al., 2025). Accordingly, AI should be positioned as a decision-support tool that narrows the candidate space and guides experimental prioritization, while final conclusions remain dependent on analytical, pharmacological, and clinical evidence. Representative phenolic compounds investigated through computational target-prediction, network-pharmacology, and AI-assisted prioritization approaches for neurodegenerative applications are summarized in Table 1.

**TABLE 1:** Representative phenolic compounds investigated through computational target-prediction, network-pharmacology, and artificial intelligence (AI)-assisted prioritization approaches for neurodegenerative applications.

Compound	Disease focus	Main predicted targets/pathways	Databases and computational tools	References
Resveratrol	AD, PD	SIRT1, AKT1, TNF, PI3K–AKT signaling	SwissTargetPrediction, TCMSP, SuperPred, SEA, STRING, KEGG	Wu et al., 2024
Curcumin	AD	BACE1, GSK3β, NF-κB, inflammatory pathways	SwissTargetPrediction, STITCH, BindingDB, TTD, OMIM, STRING, KEGG	Vijh et al., 2023
Quercetin	AD	AKT1, MAPK, axonogenesis and neuroprotection	SwissTargetPrediction, DrugBank, STRING, GO, KEGG	Wei et al., 2022
Rosmarinic acid and rosemary phenolics	AD	Apoptosis regulation, MAPK/PI3K–Akt-related pathways	PharmMapper, SwissTargetPrediction, GeneCards, OMIM, STRING, Cytoscape, GO, KEGG	Zhao et al., 2025

**FIGURE 3:**
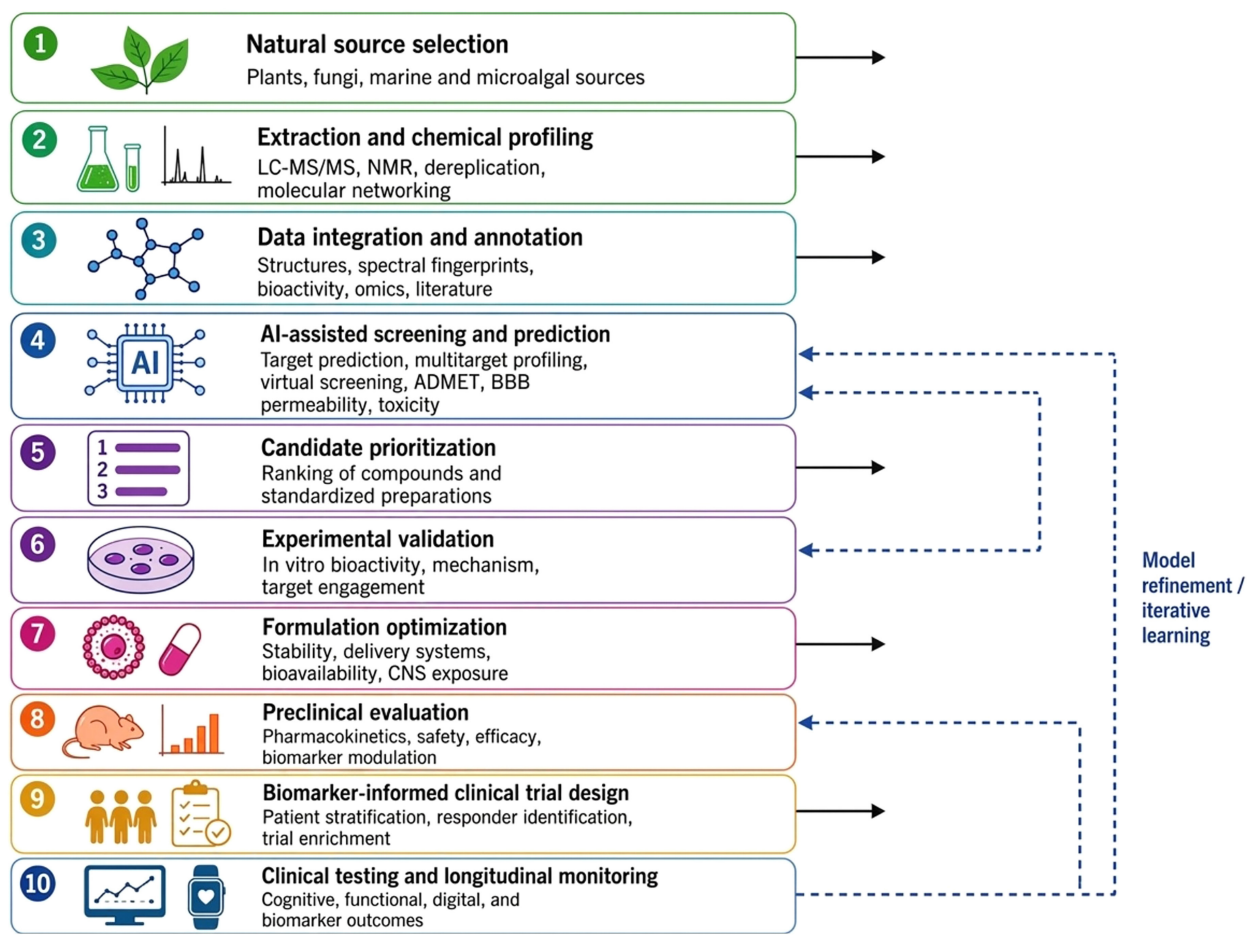
Typical workflow of artificial intelligence (AI)-assisted natural product drug discovery for neurodegenerative applications. AI and machine-learning approaches integrate chemical, biological, and clinical data to support candidate prioritization, validation, formulation, preclinical assessment, and biomarker-informed clinical development within an iterative model-refinement process.

The main stages of AI-assisted natural product discovery and translational development are summarized in Figure 3.

### 5.4 Standardization, clinical translation, and AI-supported trial design

Standardization and reproducibility remain major barriers to the clinical development of natural medicines. Botanical products and complex preparations may vary according to species, chemotype, geographic origin, cultivation and harvesting conditions, extraction, manufacturing, and storage, resulting in batch-to-batch differences in composition and biological activity (Das et al., 2024; Alum et al., 2025). Reliable translation therefore requires authenticated raw materials, comprehensive phytochemical fingerprinting, validated analytical methods, reference standards, and manufacturing controls ensuring identity, purity, potency, stability, and compositional consistency (Alum et al., 2025; European Medicines Agency, 2022a). These multidimensional quality attributes provide a further rationale for AI-assisted analysis, particularly when conventional univariate comparisons are insufficient to capture complex relationships between provenance, chemical fingerprints, manufacturing variables, and biological performance.

Regulatory frameworks issued by the European Medicines Agency (EMA) and the U.S. Food and Drug Administration (FDA) require control of source materials, manufacturing processes, specifications, contaminants, impurities, stability, safety, and efficacy which contributes to the limited number of botanical preparations that progress to full medicinal approval (European Medicines Agency, 2022a,b; U.S. Food and Drug Administration, 2020). AI and ML may complement conventional quality control by integrating chromatographic, spectroscopic, metabolomic, botanical, manufacturing, and stability data to support authentication, fingerprint classification, adulteration detection, batch-consistency assessment, and stability prediction (Mullowney et al., 2023; Zhang J. et al., 2025; Zhang K. et al., 2025). By linking chemical fingerprints with manufacturing variables and biological performance, these approaches may identify patterns that are not evident through single-marker analysis. Such tools should reinforce, rather than replace, validated analytical procedures and good manufacturing practices and require standardized inputs, traceable datasets, external validation, and interpretable outputs (Durant et al., 2024; Othman et al., 2025).

Clinical evidence for natural medicines in neurodegenerative diseases remains limited by small samples, short interventions, heterogeneous formulations and doses, variable populations, and inconsistent cognitive, functional, and biomarker endpoints. These limitations hinder replication and make it difficult to distinguish clinically meaningful or disease-modifying effects from transient symptomatic changes, confounding, or methodological variability (Goyal et al., 2024; Madhubala et al., 2024; Conti et al., 2025). They also restrict the development of reliable AI models, because algorithms trained on small, heterogeneous, or poorly standardized datasets may reproduce study-specific noise rather than identify generalizable treatment-response patterns. Traditional use and mechanistic plausibility therefore cannot substitute for adequate product characterization, controlled clinical trials, and a coherent relationship among quality, pharmacology, safety, and clinical outcomes (European Medicines Agency [EMA], 2017; U.S. Food and Drug Administration, 2020). AI-supported clinical development should consequently be based on well-characterized interventions, predefined outcomes, harmonized data collection, and sufficiently representative populations.

Future clinical development should begin with a defined mechanistic hypothesis, robust pharmacological and pharmacokinetic characterization, and confirmation that the tested preparation is chemically standardized, stable, and reproducible across batches, in accordance with guidance from the National Center for Complementary and Integrative Health (NCCIH) and the FDA (National Center for Complementary and Integrative Health [NCCIH], 2024; U.S. Food and Drug Administration, 2020). Trials should include adequate statistical power, randomization and blinding, predefined dose–response assessment, sufficient duration and follow-up, and systematic monitoring of adherence and safety. Preventive and symptomatic objectives should be distinguished explicitly, while study populations should be characterized according to disease stage, biomarker status, comorbidities, concomitant treatments, and relevant demographic variables. Cognitive and functional outcomes should be integrated with pharmacokinetic measures and biomarkers of exposure, target engagement, and biological response (National Center for Complementary and Integrative Health [NCCIH], 2024; U.S. Food and Drug Administration, 2020). Such standardized and multimodal data collection is essential not only for conventional efficacy assessment but also for the development of AI models capable of identifying reproducible treatment-response patterns and clinically meaningful patient subgroups.

AI and ML may support biomarker-informed trial enrichment, identification of biologically coherent subgroups, prediction of attrition or adherence, integration of longitudinal cognitive and digital outcomes, and detection of heterogeneous treatment responses (Hampel et al., 2023; Kourtis et al., 2019; Lott et al., 2024). In natural-product trials, these applications may be particularly useful for relating variability in product exposure, biological response, and patient characteristics to differences in cognitive or biomarker outcomes. Their clinical value should, however, be demonstrated by improved participant selection, trial efficiency, outcome prediction, or identification of reproducible responder profiles compared with conventional approaches. When algorithms influence eligibility, stratification, endpoints, or response assessment, their use should be prospectively specified and subjected to transparent reporting, calibration, external validation, subgroup and fairness analyses, and comparison with conventional trial-design approaches (Ghassemi et al., 2021; Vasey et al., 2022).

Detailed reporting is equally important because reproducibility depends on precise documentation of botanical identity, chemical composition, preparation, dosage, manufacturing, quality control, and batch characteristics. Adherence to the CONSORT extension for herbal interventions would improve transparency and comparison across studies (Gagnier et al., 2006). For AI-supported analyses, such reporting is also essential to ensure that product-related variables are encoded consistently, that datasets can be harmonized across studies, and that apparent treatment-response patterns are not driven by unrecognized differences in composition or manufacturing. The translational value of AI lies not in compensating for poorly characterized products or weak trials, but in helping convert standardized natural medicines into testable, biomarker-informed, and clinically stratified hypotheses whose benefit can be evaluated prospectively against conventional development and trial-design approaches.

## 6 Artificial intelligence in drug repurposing and personalized medicine

### 6.1 Machine learning for drug repurposing and multimodal target discovery

Machine-learning approaches may support drug repurposing and target discovery by integrating chemical structures, drug–target relationships, bioactivity profiles, omics signatures, disease annotations, clinical data, and literature-derived evidence. This is particularly relevant to neurodegenerative diseases, in which biological heterogeneity and interacting pathological pathways limit purely single-target strategies (Qiu and Cheng, 2024). Graph-based, network-medicine, and representation-learning methods may connect compounds with disease-relevant molecular states and support polypharmacology-aware prioritization. Curated natural-product resources may extend these pipelines through structural, bioactivity, and source-related metadata, although database-derived associations require experimental confirmation (Zeng et al., 2024; Qiu and Cheng, 2024).

In Alzheimer’s disease, DRIAD integrated transcriptomic perturbation signatures from human neural cells with AMP-AD brain data to rank approved or clinically tested drugs according to their association with Braak-stage pathology (Rodriguez et al., 2021). Real-world-data approaches have complemented molecular prioritization through target-trial emulation across longitudinal healthcare databases, adding an observational clinical-evidence layer to repurposing pipelines (Charpignon et al., 2022; Zang et al., 2023).

Network-based and single-cell-informed studies further illustrate the integration of target discovery and repurposing. Disease-module analyses nominated sildenafil as a potential Alzheimer’s disease modifier, with subsequent work incorporating real-world evidence and patient-derived neuronal models (Fang et al., 2021; Gohel et al., 2024). Single-cell and single-nucleus transcriptomic analyses have also identified disease-associated microglial–astrocytic networks and proposed fluticasone and mometasone as candidates (Xu et al., 2021). These examples show the importance of combining computational rankings with orthogonal molecular, experimental, and clinical evidence (Fang et al., 2021; Xu et al., 2021; Gohel et al., 2024; Cummings et al., 2025).

The principal contribution of multimodal AI is therefore to connect approved or natural-product-derived compounds with biologically meaningful targets, disease endotypes, and patient-relevant mechanisms. Repurposing signals nevertheless require prospective confirmation of mechanism, dose, central nervous system exposure, safety, target engagement, and therapeutic benefit (Qiu and Cheng, 2024; Cummings et al., 2025).

### 6.2 Personalized therapeutic strategies based on biomarkers, genetics, and clinical phenotypes

Beyond diagnosis and prognosis, AI may support therapeutic personalization by integrating biomarkers, genetics, clinical phenotype, comorbidities, medication history, functional status, and longitudinal outcomes. Such models may assist in identifying likely responders, estimating treatment-related risks, determining monitoring intensity, and updating decisions as clinical and biological trajectories evolve. Their role should remain supportive, with clinicians retaining responsibility for interpretation, shared decision-making, and final treatment selection (Myszczynska et al., 2020; Jack et al., 2024).

Alzheimer’s disease illustrates this approach because amyloid, tau, neurodegeneration, and blood-based biomarkers increasingly inform biological staging and treatment eligibility. Plasma p-tau217 may support preliminary stratification, although its interpretation depends on clinical context, assay performance, comorbidities, disease stage, and validated diagnostic workflows (Jack et al., 2024; Palmqvist et al., 2025). For anti-amyloid monoclonal antibodies, appropriate-use recommendations require biomarker confirmation, baseline MRI assessment, evaluation of vascular and hemorrhagic risk, consideration of APOE genotype in relation to amyloid-related imaging abnormalities, and continued clinical and imaging surveillance (Cummings et al., 2023; Rabinovici et al., 2025). AI-supported systems may integrate these factors with concomitant treatments and patient preferences, but predicted benefit–risk profiles require prospective validation and cannot replace specialist assessment or informed consent.

Genetic information may also support stratification in other neurodegenerative diseases. In Parkinson’s disease, variants involving GBA1, LRRK2, PRKN, SNCA, PINK1, PARK7, and VPS35 may contribute to prognosis, mechanistic classification, counseling, and eligibility for genotype- or pathway-directed trials. ML may integrate these variants with age at onset, family history, motor and non-motor features, biomarkers, and progression patterns to identify clinically coherent subgroups (Cook et al., 2024). Genetic findings should nevertheless be interpreted non-deterministically and require counseling, ancestry-aware validation, and protection against discrimination.

Clinical phenotyping remains essential because patients sharing the same molecular diagnosis may differ in cognition, neuropsychiatric symptoms, frailty, vascular burden, functional reserve, treatment tolerance, social context, and caregiver support. Useful AI systems should therefore combine molecular and clinical information and provide interpretable estimates of expected benefit, uncertainty, and safety (Ghassemi et al., 2021; Vasey et al., 2022). The objective is not automated prescribing, but a human-supervised framework in which treatment decisions are updated according to biomarker change, response, adverse events, adherence, and patient priorities. Prospective studies must determine whether AI-supported strategies improve safety, treatment appropriateness, patient-reported outcomes, equity, and net clinical benefit compared with conventional care (Vasey et al., 2022; Vickers et al., 2016).

## 7 Artificial intelligence in neurodegenerative diseases

### 7.1 AI for early detection and differential diagnosis: neuroimaging, fluid Biomarkers, Speech, and digital phenotyping

Building on the multimodal biomarker framework described in section “3.4 Multimodal biomarkers and AI-enabled preclinical detection,” AI and ML may support early detection, biological characterization, and differential diagnosis across the continuum from preclinical disease to dementia. This is particularly relevant in mild cognitive impairment, where heterogeneous trajectories cannot be reliably predicted from a single cognitive, imaging, or molecular measure. The relevant AI tasks, biomarker profiles, and progression patterns differ substantially across neurodegenerative disorders and should not be considered interchangeable (Myszczynska et al., 2020; Rossi et al., 2023). In Alzheimer’s disease, AI applications primarily focus on amyloid and tau biomarker integration, prediction of progression from mild cognitive impairment, neuroimaging-based staging, and identification of patients potentially eligible for disease-modifying treatments (Ashton et al., 2022; Cirincione et al., 2024; Jack et al., 2024; Jasodanand et al., 2025; Lee et al., 2024). In Parkinson’s disease and Parkinson’s disease dementia, disease-specific models should instead consider motor and non-motor symptoms, dopaminergic imaging, gait, speech, autonomic dysfunction, sleep-related measures, and genetic variants such as GBA1 and LRRK2 to support subtype identification and progression prediction (Postuma et al., 2015; Smith et al., 2022; Myszczynska et al., 2020). Dementia with Lewy bodies requires greater emphasis on cognitive fluctuations, visual hallucinations, REM-sleep behavior disorder, parkinsonism, autonomic dysfunction, and dopaminergic or synuclein-related biomarkers, while differential diagnosis from Alzheimer’s disease remains a major AI use case (McKeith et al., 2017; Leuzy et al., 2020; Myszczynska et al., 2020). Frontotemporal dementia is characterized by marked clinical and molecular heterogeneity, with AI applications focusing on language, behavior, executive function, structural and functional neuroimaging, and genetic or proteinopathy-related subtypes (Rascovsky et al., 2011; Myszczynska et al., 2020). Huntington’s disease differs further because genetic status is established before symptom onset, making longitudinal motor, cognitive, imaging, and digital measures especially relevant for predicting phenoconversion and progression (McColgan and Tabrizi, 2018; Myszczynska et al., 2020). Consequently, disease-specific models, endpoints, prediction horizons, and validation cohorts are required, and performance in one neurodegenerative disorder should not be assumed to generalize to another (Myszczynska et al., 2020; Rossi et al., 2023). AI should therefore be regarded as multimodal decision support rather than a stand-alone diagnostic substitute.

Neuroimaging is among the most mature application domains. Deep-learning models using structural MRI, amyloid PET, and multimodal imaging have shown promising performance in distinguishing cognitively unimpaired individuals, mild cognitive impairment, and Alzheimer’s disease (Castellano et al., 2024; Jasodanand et al., 2025). In a study of 51,269 participants from nine independent datasets, a multimodal model achieved micro-averaged AUROCs of 0.94 for classifying normal cognition, mild cognitive impairment, and dementia and 0.96 for differentiating dementia etiologies (Xue et al., 2024). Fluid biomarkers may further improve classification and progression prediction when combined with imaging, genetic, demographic, or cognitive variables, although limited longitudinal validation, interpretability, and population diversity remain important barriers (Blanco et al., 2023). High retrospective discrimination alone does not establish prospective clinical utility.

Speech analysis offers a lower-cost and ecologically relevant modality because connected speech reflects lexical retrieval, semantic organization, syntax, fluency, executive control, and memory (Smeriglio, 2024b). Speech abnormalities have been identified in cerebrospinal-fluid-defined prodromal Alzheimer’s disease, and an automated model predicted progression from mild cognitive impairment to Alzheimer’s disease within six years with 78.5% accuracy and 81.1% sensitivity (Mazzon et al., 2019; Amini et al., 2024). However, generalizability may be affected by language, education, task design, recording conditions, and population characteristics.

Digital phenotyping extends longitudinal assessment through smartphones and wearables capturing gait, sleep, mobility, activity, device interaction, and everyday function (Kourtis et al., 2019; Lott et al., 2024). These measures may detect within-person change between clinical visits, but require standardized acquisition, transparent feature extraction, management of missing data, privacy protection, and validation across devices and populations.

The most realistic role of AI is therefore to combine these complementary modalities in support of screening, biological staging, differential diagnosis, and longitudinal risk estimation (Smeriglio, 2024a; Blanco et al., 2023; Xue et al., 2024). Translation will require external and prospective validation, calibration, interpretable outputs, comparison with clinician-led assessment, and evidence that AI-supported pathways improve diagnostic timing, resource allocation, or patient outcomes beyond conventional practice (Ghassemi et al., 2021; Vasey et al., 2022). The principal multimodal biomarker domains, AI and ML techniques, clinical applications, validation status, and current translational limitations are summarized in Table 2.

**TABLE 2:** Comparison of multimodal biomarkers, artificial intelligence (AI) techniques, clinical applications, validation status, and current limitations in neurodegenerative diseases.

Biomarker/data domain	Representative inputs	AI/ML techniques	Main clinical applications	Current validation status	Main limitations
Structural neuroimaging	MRI-derived cortical thickness, hippocampal volume, regional atrophy, white-matter lesions	Support vector machines, random forests, convolutional neural networks, radiomics, representation learning	Early detection, differential diagnosis, disease staging, prediction of MCI progression	Extensive retrospective internal validation, some external cohort validation, limited prospective workflow evaluation	Scanner and site effects, segmentation variability, data leakage, limited interpretability, reduced generalizability across populations
Molecular and functional neuroimaging	Amyloid PET, tau PET, FDG-PET, functional connectivity, diffusion MRI, dopaminergic imaging	CNNs, multimodal deep learning, graph neural networks, feature-fusion models	Biological staging, differential diagnosis, treatment eligibility, progression prediction	Promising performance in selected research cohorts, external and prospective validation remains limited	High cost, limited accessibility, acquisition heterogeneity, small datasets, disease-specific applicability
Fluid biomarkers	Plasma or CSF Aβ42/40, p-tau181, p-tau217, p-tau231, NfL, GFAP, inflammatory and metabolic markers	Logistic regression, gradient boosting, random forests, support vector machines, multimodal fusion	Screening, biological classification, prognosis, trial enrichment, treatment monitoring	Increasing analytical and clinical validation for selected Alzheimer-related biomarkers, limited validation of integrated AI panels in routine care	Assay variability, preanalytical effects, threshold dependence, comorbidities, incomplete disease specificity, limited calibration across populations
Genetic and omics data	APOE, GBA1, LRRK2, polygenic risk scores, transcriptomics, proteomics, metabolomics	Elastic-net models, gradient boosting, deep neural networks, autoencoders, network-based methods	Risk stratification, subtype identification, mechanistic profiling, prediction of treatment response	Mostly retrospective or exploratory, limited external validation and clinical impact assessment	High dimensionality, small sample size, population stratification, batch effects, overfitting, limited interpretability
Cognitive and clinical measures	Neuropsychological scores, functional scales, comorbidities, medications, demographic variables	Logistic regression, decision trees, random forests, gradient boosting, survival models	Diagnosis, prognosis, progression prediction, referral and monitoring	Frequently validated internally and sometimes externally, comparatively feasible for implementation	Education and language effects, missing data, variable assessment protocols, ceiling and floor effects
Speech and language biomarkers	Acoustic features, lexical diversity, semantic coherence, pauses, prosody, discourse structure	Natural-language processing, transformers, recurrent neural networks, acoustic classifiers	Early detection, differential diagnosis, remote monitoring, identification of cognitive change	Mostly cross-sectional or small-cohort validation, limited multilingual and prospective evaluation	Language, accent, education, recording conditions, task dependence, privacy concerns
Gait, mobility, and wearable-derived measures	Gait speed, stride variability, activity, sleep, heart rate, mobility patterns, device interaction	Time-series models, random forests, deep learning, anomaly detection, digital phenotyping	Longitudinal monitoring, detection of functional decline, fall risk, treatment-response assessment	Predominantly feasibility and pilot studies, limited large-scale prospective validation	Device heterogeneity, missing data, adherence, digital divide, sensor drift, uncertain clinical thresholds
Electronic health records and real-world data	Diagnoses, medications, laboratory tests, healthcare utilization, clinical notes	Gradient boosting, NLP, deep neural networks, survival analysis	Case finding, risk prediction, referral prioritization, care-pathway optimization	Retrospective validation is common, prospective impact studies remain uncommon	Coding bias, missingness, dataset shift, confounding, limited transportability, workflow dependence
Integrated multimodal models	Combinations of imaging, fluid biomarkers, cognition, genetics, speech, wearables, and EHR data	Early, intermediate, or late fusion, multimodal transformers, ensemble learning, graph-based models	Individualized risk prediction, differential diagnosis, disease staging, progression modeling, treatment selection	Often higher internal discrimination than single-modality models, external reproducibility and prospective clinical utility remain insufficiently demonstrated	Complex missing-data patterns, asynchronous acquisition, high infrastructure requirements, limited interpretability, risk of overfitting, uncertain incremental clinical value

### 7.2 Beyond benchmark accuracy: interpretability, robustness, and clinical utility

A major limitation of current AI applications in cognitive decline is their reliance on aggregate performance metrics without sufficient evaluation of how models learn, generalize, and fail across clinically heterogeneous populations. High benchmark performance may reflect data leakage, shortcut learning, demographic imbalance, scanner effects, or site-specific acquisition patterns rather than biologically meaningful signatures of neurodegeneration (Kelly et al., 2019; Roberts et al., 2021; Wynants et al., 2020). Models should therefore be evaluated according to interpretability, subgroup robustness, calibration, failure modes, and clinical actionability in addition to discrimination performance (Sendak et al., 2020; Vollmer et al., 2020; Vasey et al., 2022).

Direct comparison across AI and ML studies remains challenging because reported performance depends on the clinical task, disease stage, class balance, prediction horizon, data modality, cohort composition, and validation design (Myszczynska et al., 2020; Vollmer et al., 2020). Conventional machine-learning models based on selected clinical, cognitive, imaging, or biomarker features may offer greater transparency and can perform competitively in small or moderately sized datasets (Rudin, 2019; Myszczynska et al., 2020). By contrast, deep-learning architectures can extract complex representations from high-dimensional imaging, speech, or multimodal data, but generally require larger samples and are more vulnerable to overfitting, site-specific effects, and limited interpretability (Myszczynska et al., 2020; Roberts et al., 2021). Multimodal models often report higher discrimination than single-modality approaches, although these gains are not always preserved in external cohorts (Cirincione et al., 2024; Jasodanand et al., 2025; Lee et al., 2024; Wang Y. et al., 2024). Moreover, most studies emphasize accuracy, sensitivity, specificity, F1 score, or area under the receiver-operating-characteristic curve, whereas calibration, subgroup robustness, decision-curve analysis, workflow impact, and prospective clinical utility are reported far less consistently (Van Calster et al., 2019; Vickers et al., 2016; Vollmer et al., 2020; Vasey et al., 2022). Thus, high benchmark performance should not be interpreted as evidence of clinical superiority in the absence of external validation, transparent model explanation, and demonstrated improvement in patient-level decisions or outcomes (Kelly et al., 2019; Ghassemi et al., 2021; Vasey et al., 2022; Vollmer et al., 2020).

External validation across institutions, devices, languages, and demographic groups is necessary to establish reproducibility, while prospective studies are required to assess model behavior within clinical workflows (Kelly et al., 2019; Sendak et al., 2020; Vasey et al., 2022). Calibration is equally important because accurate ranking of higher- and lower-risk individuals does not ensure reliable absolute-risk estimates for screening, referral, or intervention decisions (Van Calster et al., 2019; Vollmer et al., 2020).

Explainable-AI methods may help determine whether predictions are associated with plausible neuroanatomical, cognitive, biomarker, or behavioral patterns, while fairness analyses can identify systematic underperformance in underrepresented populations (Rudin, 2019; Ghassemi et al., 2021). However, post hoc explanations do not demonstrate biological validity or causality and should be interpreted alongside data provenance, sensitivity analyses, subgroup performance, uncertainty estimates, and clinically relevant failure cases.

Clinical utility requires comparison with existing assessments, validated risk scores, and clinician judgment to determine whether AI improves diagnostic timing, referrals, trial enrolment, resource allocation, intervention selection, or patient outcomes (Sendak et al., 2020; Vasey et al., 2022). Decision-curve analysis, prospective impact studies, workflow evaluation, and assessment of false-positive and false-negative consequences may provide more clinically informative evidence than discrimination metrics alone (Vickers et al., 2016; Vasey et al., 2022). Clinically useful AI should therefore function as transparent, human-supervised decision support whose adoption depends on demonstrable added value, appropriate communication of uncertainty, and evidence of safety, equity, feasibility, and benefit in the intended setting (Ghassemi et al., 2021; Vasey et al., 2022). Overall, no single model class appears consistently superior across clinical settings. Models with slightly lower discrimination but better calibration, interpretability, external reproducibility, and workflow compatibility may ultimately provide greater clinical value than more complex systems optimized primarily for benchmark accuracy.

### 7.3 Risk stratification and prediction of disease progression

AI-supported risk stratification extends beyond diagnostic classification by integrating cognitive, imaging, fluid-biomarker, genetic, demographic, and longitudinal data to estimate disease stage, likelihood and timing of progression, and individual risk. Such models may distinguish relatively stable individuals from faster progressors and identify patients who could benefit from confirmatory testing, specialist monitoring, clinical-trial enrolment, or earlier intervention (Hampel et al., 2023; Vaghari et al., 2025). Multimodal approaches have shown promising results in predicting conversion from mild cognitive impairment to Alzheimer’s disease and modeling longer-term trajectories, although clinical use requires external and prospective validation, calibration, clearly defined prediction horizons, and demonstrated improvement over conventional assessment (Lee et al., 2024; Wang Y. et al., 2024; Cirincione et al., 2024; Vaghari et al., 2025).

Blood-based biomarkers further strengthen the potential for scalable stratification. Plasma p-tau217 shows high diagnostic accuracy for Alzheimer’s disease pathology, and in 2025 the FDA cleared the Lumipulse G pTau217/β-Amyloid 1-42 Plasma Ratio to aid diagnosis in adults aged 50 years or older with signs or symptoms of cognitive decline in specialized-care settings (U.S. Food and Drug Administration, 2025; Palmqvist et al., 2025). Because the assay is neither a population-screening nor a stand-alone diagnostic test, results must be interpreted with clinical information (U.S. Food and Drug Administration, 2025). AI may therefore be most useful for combining blood biomarkers with cognitive, imaging, vascular, demographic, and longitudinal data rather than treating an isolated result as determinative.

The translational objective is to move from static diagnosis toward dynamic prognosis. Clinically meaningful models should communicate prediction horizon, uncertainty, contributing variables, and the likely consequences of alternative clinical actions. These estimates may support trial enrichment, confirmatory testing, individualized monitoring, and preventive or therapeutic planning. Adoption nevertheless requires robust subgroup performance, interpretable outputs, calibration across relevant time horizons, and evidence of added clinical utility in prospective workflows (Ghassemi et al., 2021; Vasey et al., 2022; Van Calster et al., 2019; Vickers et al., 2016).

### 7.4 Monitoring disease trajectories and supporting early clinical decision-making

Beyond diagnosis, AI may support longitudinal monitoring by integrating repeated cognitive assessments, biomarkers, imaging, and real-world behavioral data. Such systems may detect within-person change, update progression estimates, and signal when further assessment or modification of care is appropriate. Their role should remain that of human-supervised decision support rather than autonomous decision-making (Lott et al., 2024; Vaghari et al., 2025; Vasey et al., 2022). European initiatives such as PROMINENT and AI-Mind illustrate the transition from single-time-point classification toward multimodal and workflow-oriented support for suspected cognitive impairment and mild cognitive impairment (Alzheimer Europe, 2025b,e).

AI may also personalize non-pharmacological interventions by adapting task difficulty, feedback, frequency, and content to individual performance. Computer-based cognitive training and virtual-reality interventions may support memory, orientation, and functional skills, although outcomes remain heterogeneous and these approaches should complement conventional rehabilitation (García-Casal et al., 2017; Pedroli et al., 2018; Costanzo et al., 2023; Tortora et al., 2024).

Wearable sensors, smart-home systems, and connected devices may monitor mobility, sleep, inactivity, falls, wandering, routine disruption, and behavioral change, potentially enabling earlier intervention and greater continuity of care (Rashidi and Cook, 2009; Husebo et al., 2020; Asiri et al., 2023). Their real-world value depends on reliable detection, acceptable false-alert rates, interoperability, privacy, informed consent, and user acceptability. Participatory initiatives such as AI4HOPE and Alzheimer Europe’s public-involvement activities emphasize the importance of co-design with people living with dementia and caregivers (Alzheimer Europe, 2025c,d).

Digital tools may further assist caregivers through telehealth, education, reminders, communication support, and access to monitoring information. Tele-STAR, voice assistants, and socially assistive technologies such as PARO have shown feasibility or benefits for selected caregiver, engagement, mood, or agitation outcomes, although evidence remains heterogeneous (Moyle et al., 2017; Lindauer et al., 2019; Nunez et al., 2025; Salai et al., 2022). These tools should support, rather than replace, professional oversight and person-centered relationships (Kitwood, 1997; Brooker, 2004).

Prospective workflow studies should determine whether longitudinal AI systems detect meaningful change earlier than conventional follow-up and improve clinical action, workload, quality of life, and outcomes relevant to patients and caregivers. Evaluation should include alert relevance, clinician response, adherence, equity, privacy, and downstream care consequences rather than predictive accuracy alone (Ghassemi et al., 2021; Vasey et al., 2022).

### 7.5 Ethical, regulatory, and implementation challenges in AI-supported dementia care

Artificial intelligence-supported dementia care raises distinctive ethical and organizational challenges because algorithm-informed decisions may affect diagnosis, prognosis, treatment eligibility, autonomy, and future planning. Moreover, decision-making capacity may change during disease progression, increasing the importance of accessible communication, person-centered care, and effective human oversight. AI should therefore support, rather than replace, neurological, neuropsychological, and multidisciplinary judgment (Kitwood, 1997; Brooker, 2004; World Health Organization, 2021).

According to the World Health Organization (WHO) transparency, accountability, privacy, and equity are central requirements. WHO guidance emphasizes that ethics and human rights should be embedded throughout the design, validation, and deployment of health-related AI, while unrepresentative datasets and age-biased assumptions may amplify inequalities affecting older adults (World Health Organization, 2021, 2022, World Health Organization., 2024). Dementia-specific concerns include informed consent, evolving decision-making capacity, domestic or institutional surveillance, caregiver access to data, and the risk that technology may undermine dignity or reduce human contact (Alzheimer Europe, 2013; Alzheimer’s Disease International, 2019; Swaffer, 2014; Smeriglio, 2025). Participatory design involving people living with dementia and caregivers is therefore essential (Alzheimer Europe, 2026).

Algorithmic bias should be addressed throughout the model lifecycle rather than assessed only after deployment (Kelly et al., 2019; Vollmer et al., 2020; Vilor-Tejedor et al., 2026). Bias may arise from underrepresentation of older adults, ethnic and linguistic minorities, individuals with lower educational attainment, rural populations, or patients with multiple comorbidities, as well as from differences in diagnostic access, device use, and data completeness (Livingston et al., 2024; Vilor-Tejedor et al., 2026). Development and validation should therefore include subgroup-specific performance analyses, calibration assessment, investigation of proxy variables, and predefined mitigation strategies when systematic disparities are identified (Van Calster et al., 2019; Vollmer et al., 2020; Vasey et al., 2022).

Patient privacy and data governance are particularly important because dementia-related AI may combine sensitive clinical, genetic, imaging, behavioral, wearable, and home-monitoring data (Kourtis et al., 2019; Lott et al., 2024; Vilor-Tejedor et al., 2026). Governance frameworks should define lawful data access, informed consent and re-consent procedures, data minimization, purpose limitation, retention periods, secure storage, secondary use, data sharing, and the rights of patients and authorized caregivers (European Union, 2024; Alzheimer Europe, 2026; Vollmer et al., 2020). Particular safeguards are required when decision-making capacity changes over time or when passive monitoring generates data about household members who have not directly consented (Alzheimer Europe, 2013, 2026; Vilor-Tejedor et al., 2026).

Transparency should extend beyond technical explainability (Ghassemi et al., 2021; Vollmer et al., 2020). Patients, caregivers, and clinicians should receive understandable information on the intended use of the system, the data on which predictions are based, the degree of uncertainty, known limitations, and the consequences of acting—or not acting—on an algorithmic recommendation (Alzheimer Europe, 2026; Vasey et al., 2022; Vilor-Tejedor et al., 2026). Accountability must also remain clearly allocated: developers are responsible for model design and performance documentation, healthcare institutions for deployment and monitoring, and clinicians for contextual interpretation and final decisions (European Union, 2024; Kelly et al., 2019; Vollmer et al., 2020). Explicit audit trails, incident-reporting procedures, mechanisms for contesting or reviewing recommendations, and clear liability arrangements are therefore essential when AI contributes to diagnosis, prognosis, treatment eligibility, or care planning (European Union, 2024; Alzheimer Europe, 2026; Vilor-Tejedor et al., 2026).

Responsible implementation also requires representative datasets, external and prospective validation, calibration, interpretable outputs, and monitoring of bias and performance drift across clinical settings (Shankar et al., 2025; Breithaupt et al., 2025; Ghassemi et al., 2021; Vasey et al., 2022). Clinicians must understand a model’s intended use, uncertainty, limitations, and clinically relevant failure modes, particularly when predictions influence treatment, monitoring, autonomy, or family planning.

Under the European Union AI Act, healthcare applications may be subject to high-risk requirements concerning risk management, technical documentation, data governance, transparency, performance monitoring, and human oversight (European Union, 2024). Clinical deployment additionally requires interoperability, cybersecurity, institutional accountability, staff training, workflow integration, and explicit procedures for reviewing or overriding algorithmic recommendations. Most dementia-related AI systems nevertheless remain at retrospective-validation, feasibility, or pilot-implementation stages rather than demonstrating effectiveness at scale in routine practice (Breithaupt et al., 2025; Steijger et al., 2025; Vilor-Tejedor et al., 2026).

Real-world implementation also depends on regulatory approval pathways, procurement and reimbursement mechanisms, and the availability of adequate healthcare infrastructure (Kelly et al., 2019; Vollmer et al., 2020; Vilor-Tejedor et al., 2026). Regulatory clearance should define the intended use, target population, required level of human oversight, performance-monitoring obligations, and procedures for managing software updates or model drift (European Union, 2024; Vasey et al., 2022; Vilor-Tejedor et al., 2026). Even technically valid systems may fail to achieve routine adoption if reimbursement does not cover acquisition, integration, clinician time, confirmatory testing, and long-term maintenance (Kelly et al., 2019; Vilor-Tejedor et al., 2026). Economic evaluation should therefore consider cost-effectiveness, budget impact, downstream diagnostic and treatment consequences, and whether AI-supported pathways reduce or increase overall resource use (Vollmer et al., 2020; Vilor-Tejedor et al., 2026). Feasibility is also likely to vary substantially across healthcare settings (Hampel et al., 2023; Teunissen et al., 2022; Vilor-Tejedor et al., 2026). In highly specialized centers, the costs of AI implementation may be partly absorbed by existing imaging, biomarker, electronic-health-record, and data-science infrastructures, whereas community-based services and resource-limited settings may face greater barriers related to software acquisition, interoperability, connectivity, workforce training, technical support, and access to confirmatory testing (Hampel et al., 2023; Lott et al., 2024; Vilor-Tejedor et al., 2026). Cost-effectiveness analyses should therefore be context-specific and adopt appropriate time horizons and perspectives, including healthcare-system, societal, patient, and caregiver costs (Livingston et al., 2024; Vilor-Tejedor et al., 2026). They should compare AI-supported precision-prevention pathways with usual care and simpler risk-stratification strategies, while accounting for implementation, maintenance, model updating, false-positive investigations, delayed or avoided diagnoses, treatment-related costs, caregiver burden, and potential reductions in institutionalization or healthcare utilization (Livingston et al., 2024; Vollmer et al., 2020; Vilor-Tejedor et al., 2026). Implementation further requires interoperable electronic health records, secure data storage, reliable connectivity, technical support, multidisciplinary expertise, and equitable access across tertiary centers, community services, and resource-limited settings (Hampel et al., 2023; Lott et al., 2024; Teunissen et al., 2022; Vilor-Tejedor et al., 2026). Without these organizational, economic, and infrastructural conditions, even accurate and well-validated AI systems are unlikely to produce sustained improvements in routine dementia prevention and care (Kelly et al., 2019; Vollmer et al., 2020; Vilor-Tejedor et al., 2026).

The relevant question is therefore whether AI can improve dementia care safely, equitably, and while preserving dignity and personhood. A human-in-the-loop model should maintain clinician accountability, communicate uncertainty appropriately, and ensure meaningful participation of patients and caregivers (Kitwood, 1997; Brooker, 2004; World Health Organization, 2021; Alzheimer Europe, 2026). Adoption should depend on demonstrable benefit, proportional data use, accessible governance, and protection against automation bias, exclusion, and excessive surveillance (Smeriglio, 2026; Vilor-Tejedor et al., 2026). The transition from conventional dementia care to human-supervised AI-supported care and its principal implementation requirements are summarized in Table 3.

**TABLE 3:** Transition from traditional dementia care to human-supervised artificial intelligence (AI)-supported care.

Dimension	Traditional care	Human-supervised AI-supported care	Implementation requirement
Clinical reasoning	Clinician interpretation of history, examination, and selected tests	Clinician integrates multimodal algorithmic outputs with clinical context	Human oversight and protection against automation bias
Diagnosis and risk estimation	Sequential assessment based on phenotype and selected biomarkers	Probabilistic integration of cognition, imaging, fluid biomarkers, and digital measures	External validation, calibration, and interpretability
Longitudinal monitoring	Periodic visits and caregiver reports	Repeated or continuous monitoring through digital platforms and sensors	Privacy, consent, alert relevance, and data governance
Intervention planning	Predominantly standardized recommendations periodically reviewed	Potentially adaptive interventions informed by changing risk and response profiles	Prospective evidence of clinical benefit and equity
Clinician role	Primary interpreter and decision-maker	Accountable supervisor of AI-assisted decision support	Training, workflow integration, and defined override procedures
Patient and caregiver involvement	Consent and person-centered care within conventional encounters	Participation in design, governance, interpretation, and care planning	Accessibility, digital literacy, and preservation of autonomy and dignity
Governance	Confidentiality, professional accountability, and clinical regulation	Additional requirements for transparency, fairness, cybersecurity, monitoring, and human oversight	Clear institutional and regulatory responsibility
Financing and reimbursement	Conventional service-based reimbursement	Additional costs for software, integration, monitoring, and staff time	Reimbursement pathways, cost-effectiveness, and budget-impact assessment
Infrastructure	Standard clinical IT and referral pathways	Interoperable multimodal data systems and continuous technical support	Secure connectivity, EHR integration, maintenance, and equitable access

## 8 Conclusion and future perspectives

The convergence of biologically informed diagnostic frameworks, modifiable-risk research, multidomain prevention, natural-product pharmacology, digital monitoring, and artificial intelligence supports a transition from predominantly reactive care toward earlier, more individualized, and prevention-oriented management of neurodegenerative diseases. Pathological and functional changes may emerge years before overt dementia, creating a potential window for biomarker-guided risk reduction, monitoring, and timely intervention (Jack et al., 2024; Livingston et al., 2024). Early intervention should not imply indiscriminate screening or medicalization of asymptomatic individuals, but should be based on validated biomarkers, individualized risk profiles, clearly defined prediction horizons, and careful interpretation of uncertainty within specialist-led pathways (Teunissen et al., 2022; Vasey et al., 2022). A first research priority is therefore to define clinically meaningful target populations, prediction horizons, and intervention thresholds through prospective studies that compare AI-supported risk stratification with established clinical and biomarker-based approaches.

Artificial intelligence may support this transition by integrating the multimodal biological, clinical, behavioral, environmental, and digital information discussed throughout this review. Such integration may improve risk stratification, longitudinal monitoring, intervention personalization, and identification of clinically meaningful disease trajectories (Myszczynska et al., 2020; Kourtis et al., 2019; Lott et al., 2024). A second research priority is to establish prospective, longitudinal, and demographically representative multimodal cohorts in which biological, clinical, behavioral, environmental, and digital measures are collected through harmonized protocols and aligned across clinically relevant time points. These studies should determine which combinations of modalities provide incremental predictive value over simpler clinical or biomarker-based models and should explicitly address missing, asynchronous, and site-dependent data. Within this framework, lifestyle and multidomain interventions provide actionable strategies for modifying vascular, metabolic, behavioral, sensory, and psychosocial risk, whereas standardized natural compounds may offer complementary candidates acting on interconnected inflammatory, oxidative, metabolic, vascular, proteostatic, synaptic, and microbiota-related pathways (Ngandu et al., 2015; Cui et al., 2023; Nahar et al., 2025). AI may further support compound and formulation prioritization, responder identification, biomarker-informed trial enrichment, and longitudinal evaluation of intervention effects (Mullowney et al., 2023; Hampel et al., 2023; Zhang J. et al., 2025; Zhang K. et al., 2025). These applications should be tested in prospectively designed intervention studies that predefine candidate-selection criteria, responder definitions, target-engagement biomarkers, adherence measures, and cognitive or functional endpoints, and that compare AI-guided personalization with standard intervention strategies.

However, neither biological plausibility nor computational performance is sufficient to establish clinical value. Natural medicines require reproducible chemical characterization, adequate bioavailability and brain exposure, target-engagement and safety assessment, standardized formulations, and evidence from adequately powered clinical trials (Goyal et al., 2024; U.S. Food and Drug Administration, 2020; National Center for Complementary and Integrative Health [NCCIH], 2024). Likewise, AI systems require representative and standardized datasets, external and prospective validation, calibration, interpretable and clinically explainable outputs, equitable subgroup performance, secure data governance, interoperability, and clearly defined human oversight (Ghassemi et al., 2021; Vasey et al., 2022; Van Calster et al., 2019; European Union, 2024). A third research priority is therefore to conduct multisite implementation studies in which model performance is evaluated across institutions, devices, languages, demographic groups, and healthcare settings. These studies should prospectively assess calibration, subgroup performance, model drift, missing-data robustness, false-positive and false-negative consequences, clinician–algorithm interaction, and predefined procedures for human override and model updating. Emerging environmental variables, including micro- and nanoplastic exposure, may eventually contribute to exposome-informed risk models, but currently require standardized measurement, longitudinal evidence, and causal validation before becoming clinically actionable predictors (Finch and Kulminski, 2019; Nihart et al., 2025; Araújo et al., 2025; Zheng et al., 2024). Accordingly, such variables should initially be evaluated in hypothesis-driven longitudinal studies using harmonized exposure measurements and should not be incorporated into clinical decision tools until they provide reproducible incremental value beyond established risk factors and biomarkers.

Future research should therefore move from fragmented evidence and retrospective model development toward prospective, longitudinal, population-diverse, and clinically embedded studies. A fourth research priority is to evaluate AI-supported pathways in pragmatic or randomized implementation trials against clearly defined comparators, including usual care, conventional risk scores, single-modality biomarker strategies, or non-personalized interventions. These studies should predefine clinically meaningful endpoints, including diagnostic timing, preventive targeting, treatment appropriateness, adherence, adverse events, cognitive and functional trajectories, quality of life, caregiver burden, healthcare utilization, cost-effectiveness, and net clinical benefit (World Health Organization, 2021; Vickers et al., 2016; Vasey et al., 2022). Research protocols should also specify implementation outcomes such as clinician acceptance, patient understanding, workflow burden, interoperability, and the resources required for model maintenance and updating. The most credible future direction is a human-supervised precision-neurology framework in which AI connects biological, clinical, behavioral, environmental, and social information with standardized and measurable interventions, while clinicians and patients retain responsibility for interpreting uncertainty, balancing expected benefits and harms, and defining meaningful care priorities. Progress should therefore be judged not by gains in benchmark accuracy alone, but by prospectively demonstrated improvements in clinical decisions, patient- and caregiver-relevant outcomes, equity, feasibility, and sustainability. Only under these conditions can AI become a clinically actionable component of equitable and person-centered prevention and management of cognitive decline and neurodegenerative diseases.
